# A Case of Inadequately Treated Bacterial Pneumonia Leading to Bronchopleural Fistula

**DOI:** 10.7759/cureus.63505

**Published:** 2024-06-30

**Authors:** Zhongqian Lin, Kevin S Kurian, Yvonne Ng, Masahiro Yabe, Anthony G Saleh

**Affiliations:** 1 Internal Medicine, New York-Presbyterian Brooklyn Methodist Hospital, Brooklyn, USA; 2 Pulmonary and Critical Care Medicine, New York-Presbyterian Brooklyn Methodist Hospital, Brooklyn, USA

**Keywords:** pulmonary decortication, bilateral pneumothoraces, aspergillus empyema, pseudomonas empyema, bronchopulmonary fistula

## Abstract

Bronchopulmonary fistula (BPF) is an abnormal connection between the pleural space and bronchial tree, potentially leading to fatal outcomes if untreated. While BPF commonly arises following lung surgery, it can also be linked to infections. This report details the case of a 47-year-old male with recent untreated bacterial pneumonia, who developed bilateral pneumothoraces with persistent air leaks, Pseudomonas and *Aspergillus *empyema, culminating in a right-sided BPF necessitating video-assisted thoracic surgery (VATS) decortication. The agenda of this presentation is to enhance early recognition of BPF, which can be presented subtly, to avert severe complications.

## Introduction

Bronchopulmonary fistula (BPF) refers to an abnormal connection between the pleural space and either the main stem, lobar, or segmental bronchus. When involving lung parenchyma, it is termed alveolo-pleural fistula. The most frequent cause of BPF is lung resection, particularly pneumonectomy (up to 4.5% incidence) [1]. Other less common causes include chest trauma, specific chemotherapies post-lung cancer surgery, and radiation therapy [2]. Inflammatory processes and pulmonary infections rarely contribute to BPF formation, as illustrated in the case of a 47-year-old male with untreated bacterial pneumonia progressing to BPF [3].

## Case presentation

**Figure 1:**
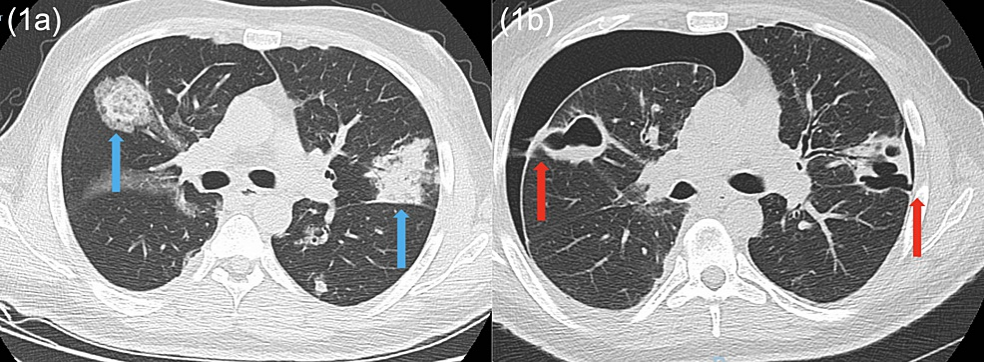
(1a) Pulmonary infiltrates from recent hospitalization without BPF; (1b) bilateral BPF where the bronchi formed sinus tracts with the pleural spaces from the current case

A 47-year-old male former heavy smoker with a past medical history of asthma and type 1 diabetes mellitus, who had been hospitalized one week ago for respiratory syncytial virus (RSV) infection with superimposed secondary bacterial pneumonia, presented with new shortness of breath and left-sided pleuritic chest pain for two days. The patient was initially tachycardic to the 120s bpm, hypoxemic to the high 80s (%) on ambient air, and had leukocytosis to 39,000 10^3/ul, as well as development of a new large left pneumothorax with mild rightward tracheal deviation, patchy opacities throughout the right upper and lower lung fields, and blebs/bullous emphysema on chest X-ray (CXR). Subsequently, a left Wayne Pigtail catheter was placed, in addition to broad-spectrum antibiotics and supplemental oxygen via a non-rebreather (NRB) mask. On hospital day 3 of his respiratory care unit stay, the patient’s chest pain became bilateral, and he was found to have a new right pneumothorax. A right chest tube was then placed. Of note, the patient’s symptoms persisted, and bilateral chest instruments had intermittent air leaks for the first week of his stay. In comparison to his prior imaging from recent hospitalization showing pulmonary infiltrates only (Figure 1a), a CT scan of the chest performed on hospital day 9 revealed BPF on the right side, as well as extensive cavitary opacities, some of which were located close to the visceral pleura, extending into the pleural space of the right lower lobe (Figure 1b). The infectious workup was concerning for right empyema with multi-resistant *Pseudomonas aeruginosa* and *Aspergillus *growth in pleural fluid and sputum cultures. The antimicrobial regimen was modified to ceftolozane-tazobactam and voriconazole. Given concern for developing trapped lung physiology and no major improvement over the second week of hospitalization, a right decortication and washout of the lung via video-assisted thoracic surgery (VATS) were performed on hospital day 14, with complete evacuation of two large pockets of pus. The patient’s bilateral pneumothoraces and infection continued to improve after the procedure and eventually resolved, and he was discharged on hospital day 30.

## Discussion

BPF is an abnormal connection between the pleural space and the bronchial tree, which carries a significant mortality rate (16-72%) if left untreated [4,5]. BPF itself is uncommon and is most often noticed post-lung cancer resection, such as pneumonectomy, and even more rarely associated with lung infections, such as pulmonary tuberculosis [6-8]. In our case, the patient had no known lung malignancy and had never undergone thoracic procedures in the past, but had a recent bacterial pneumonia superimposed on a viral infection, which was likely inadequately treated. Initially, the patient had multi-resistant Pseudomonas and was later found to have *Aspergillus *growing in the pleural fluid.

Untreated pneumonia, particularly when superimposed on structural abnormalities like blebs, can pose an increased risk for the development of BPF. Structural abnormalities such as blebs, which our patient exhibited, are small air-filled sacs on the lung’s surface that can weaken the lung tissues and likely increase the likelihood of forming BPF. In addition, the patient’s extensive smoking history and his diabetes may also have contributed to his clinical presentation [9].

The clinical features of BPF vary from acute symptoms of tension pneumothorax to subacute symptoms of empyema. However, it has also been reported that sometimes the presentation could be very subtle, such as only an air leak from the chest tube [10]. This is similar to our patient’s clinical scenario. Although our patient had both bilateral pneumothoraces and empyema, he did not exhibit any significant hemodynamic instability, fever, or sputum production, but did have an intermittent persistent air leak in his chest tube drainage system.

## Conclusions

Given the high mortality rate of BPF, it is crucial for physicians to recognize its causes, risk factors, and presentations. Although most cases of BPF are associated with pulmonary surgery, other conditions, such as pneumonia and empyema, which are common diagnoses for hospital admissions, can also increase the risk. This case highlights the need for heightened awareness among clinicians when encountering patients with unresolved pneumothorax after chest tube insertion or with unexplained persistent air leaks in the chest tube system. Overall, the case underscores the importance of prompt recognition and diagnosis of BPF to facilitate early invasive interventions, such as decortication via VATS, thereby improving patient outcomes.

## References

[1] Cusumano G, Alifano M, Lococo F (2019). Endoscopic and surgical treatment for bronchopleural fistula after major lung resection: an enduring challenge. J Thorac Dis.

[2] van de Pas JM, van Roozendaal LM, Wanders SL, Custers FL, Vissers YL, de Loos ER (2020). Bronchopleural fistula after concurrent chemoradiotherapy. Adv Radiat Oncol.

[3] Shin K, Hifumi T, Tsugitomi R, Isokawa S, Shimizu M, Otani N, Ishimatsu S (2021). Empyema with fistula successfully treated with a comprehensive approach including bronchial blocker and embolization receiving veno-venous extracorporeal membrane oxygenation. Acute Med Surg.

[4] Liang J, Field A, Liang K (2013). Bronchopleural fistula. West J Emerg Med.

[5] Salik I, Vashisht R, Abramowicz AE (2024). Bronchopleural fistula. StatPearls [Internet].

[6] Clark JM, Cooke DT, Brown LM (2020). Management of complications after lung resection: prolonged air leak and bronchopleural fistula. Thorac Surg Clin.

[7] Nagahiro I, Aoe M, Sano Y, Date H, Andou A, Shimizu N (2007). Bronchopleural fistula after lobectomy for lung cancer. Asian Cardiovasc Thorac Ann.

[8] Bathobakae L, Shahid A, Wilkinson T (2023). Tuberculous bronchopleural fistula: a rare and life-threatening disease. J Investig Med High Impact Case Rep.

[9] Li SJ, Fan J, Zhou J, Ren YT, Shen C, Che GW (2016). Diabetes mellitus and risk of bronchopleural fistula after pulmonary resections: a meta-analysis. Ann Thorac Surg.

[10] Leuzzi G, Facciolo F, Pastorino U, Rocco G (2015). Methods for the postoperative management of the thoracic oncology patients: lessons from the clinic. Expert Rev Respir Med.

